# Up-regulation of avian uncoupling protein in cold-acclimated and hyperthyroid ducklings prevents reactive oxygen species production by skeletal muscle mitochondria

**DOI:** 10.1186/1472-6793-10-5

**Published:** 2010-04-28

**Authors:** Benjamin Rey, Damien Roussel, Caroline Romestaing, Maud Belouze, Jean-Louis Rouanet, Dominique Desplanches, Brigitte Sibille, Stéphane Servais, Claude Duchamp

**Affiliations:** 1 Université de Lyon, F-69000, Lyon; Laboratoire de Physiologie Intégrative, Cellulaire et Moléculaire, CNRS - UMR 5123 Université Lyon 1, 43 Bvd 11 Novembre 1918, F-69622 Villeurbanne Cedex, France

## Abstract

**Background:**

Although identified in several bird species, the biological role of the avian homolog of mammalian uncoupling proteins (avUCP) remains extensively debated. In the present study, the functional properties of isolated mitochondria were examined in physiological or pharmacological situations that induce large changes in avUCP expression in duckling skeletal muscle.

**Results:**

The abundance of avUCP mRNA, as detected by RT-PCR in gastrocnemius muscle but not in the liver, was markedly increased by cold acclimation (CA) or pharmacological hyperthyroidism but was down-regulated by hypothyroidism. Activators of UCPs, such as superoxide with low doses of fatty acids, stimulated a GDP-sensitive proton conductance across the inner membrane of muscle mitochondria from CA or hyperthyroid ducklings. The stimulation was much weaker in controls and not observed in hypothyroid ducklings or in any liver mitochondrial preparations. The production of endogenous mitochondrial reactive oxygen species (ROS) was much lower in muscle mitochondria from CA and hyperthyroid ducklings than in the control or hypothyroid groups. The addition of GDP markedly increased the mitochondrial ROS production of CA or hyperthyroid birds up to, or above, the level of control or hypothyroid ducklings. Differences in ROS production among groups could not be attributed to changes in antioxidant enzyme activities (superoxide dismutase or glutathione peroxidase).

**Conclusion:**

This work provides the first functional *in vitro *evidence that avian UCP regulates mitochondrial ROS production in situations of enhanced metabolic activity.

## Background

The respiratory chain function of mitochondria constitutes a major cellular source of superoxide and derived reactive oxygen species (ROS) *in vivo *[1,2]. ROS, including hydroxyl and hydroperoxyl radicals, can alter mitochondrial and cellular lipid membranes, proteins and nucleic acids and may be implicated in various pathological diseases, accelerated senescence and aging [2,3]. Different protective and adaptive responses can prevent an imbalance between oxidant production and antioxidant capacities, eventually repair oxidative damage and thereby limit the deleterious effects of ROS [2,4]. These responses include several powerful antioxidant enzymes such as superoxide dismutase (SOD), catalase and glutathione peroxidase. Nevertheless, other mechanisms for reducing ROS production (especially in response to metabolic stress) at the level of the mitochondrial respiratory chain may be of particular importance to limit oxidative stress.

Uncoupling proteins (UCPs) belong to a family of mitochondrial proteins that may cause a leak of protons across the inner membrane, thus uncoupling the oxidation of reduced substrates from the phosphorylation of ADP to ATP [5]. Although the major physiological function of mammalian UCP1 (brown fat-specific) is adaptive thermogenesis [6,7], the roles of the other mammalian UCPs, UCP2 (ubiquitous) and UCP3 (skeletal muscle and adipose tissue), are still a matter of debate. It was proposed that the proton leak activity of these more ubiquitous UCPs would result in a mild uncoupling that would decrease proton motive force and stimulate oxygen consumption, thus reducing local oxygen tension and attenuating superoxide production [8-10]. Mild uncoupling of oxidative phosphorylation was indeed shown to markedly reduce superoxide production through various mechanisms [8,10-13]. A role for UCP2 and UCP3 in attenuating superoxide production by the electron transport chain to protecting against oxidative damage is supported by the increased cellular production of ROS in UCP2 knockout mice [14] and the increased mitochondrial oxidative damage in mice underexpressing UCP3 [15]. Further, inhibition of UCPs by nucleotides such as GDP increased both proton motive force and the mitochondrial production of ROS [16,17].

The activity of mammalian UCPs is tightly regulated. For instance, at physiological nucleotide concentrations the activity of UCPs closely depends on appropriate activators [3,9] such as free fatty acids, which are well-known physiological activators of UCP1 ([6] for review). Activators of other UCPs are not as well characterized, but it was shown *in vitro *that superoxide [18] or oxidized derivatives of fatty acids such as 4-hydroxy-2-nonenal [19] are potent activators of mammalian UCP1, UCP2 and UCP3 as well as plant [20] and avian UCPs [21]. The use of these activators has thus been considered a relevant method by which to reveal the activity of functional UCPs *in vitro *[18].

Despite increasing amounts of literature in this field, the physiological role of the unique avian UCP [22] remains controversial. Since its first description by Raimbault and coworkers in 2001, its role was putatively associated with facultative thermogenesis due to evidence of increased UCP expression after cold acclimation, glucagon treatment or hyperthyroidism (all situations leading to an increase in metabolic rate and a rise in thermogenic capacity) [23-26] and on the basis of its down regulation after prolonged fasting [27]. However, the thermogenic function of the avian UCP has been highly questioned in recent literature [28]. Avian UCP has also been thought to play a role in the control of lipid metabolism [29] but may also confer preventive effect against free radical damage [30-32]. Indeed, previous works showed a correlative reduction in mitochondrial ROS production with an over expression of the avUCP gene in short-term fasted chickens [33]. Conversely, a mitochondrial overproduction of ROS is associated with a down regulation of avUCP in acutely heat stressed chickens [34]. However, to date, the unique functional result comes from yeast cells and shows that avUCP overexpression in yeast triggers protection against the deleterious impact of ROS [30].

The aim of this work was to more fully assess the physiological role of avUCP as a putative antioxidant mechanism. We used chronic cold acclimation and pharmacological manipulations of thyroid status to induce large variations in UCP expression in duckling tissues. We then investigated the functional properties (respiration, proton leak and ROS production) of skeletal muscle and liver mitochondria *in vitro *after activation/inhibition of avUCP. We show a strong correlation between UCP-induced proton leak activation and a GDP-sensitive ROS production by mitochondria from tissues expressing avUCP. Our data clearly support the importance of the avian uncoupling protein in the prevention of mitochondrial ROS production and oxidative stress in birds in situations of intense metabolic activity.

## Methods

### Animals

Birds were cared for under the French Code of Practice for the Care and Use of Animals for Scientific Purposes.

Thirty-seven male Muscovy ducklings (*Cairina moschata *L, pedigree R31, Institut National de la Recherche Agronomique) were obtained just after hatching from a commercial stock breeder (Ets Grimaud, France). Newly hatched ducklings were kept at thermoneutrality under a constant photoperiod (10:14 h light-dark cycle). They were fed *ad libitum *with a commercial mash (Genthon 5A, Genthon, France) and had free access to water. When the ducklings were one week old, they were randomly assigned to one of four experimental groups and kept for four more weeks.

One group was kept at thermoneutrality (25°C) from that age and constituted the thermoneutral (TN) control group (n = 12). The second group was reared in the cold (4°C) and constituted the cold-acclimated (CA) group (n = 11). This cold acclimation schedule was shown to stimulate the development of skeletal muscle non-shivering thermogenesis by five weeks of age [35,36]. The third group of ducklings, referred to as hypothyroid (hypo, n = 7), was kept at thermoneutrality and their thyroid status was pharmacologically altered by giving them 6-n-propyl-2-thiouracil (PTU; Sigma; 0.15% w/v) in drinking water. PTU is well known to block thyroperoxidase and peripheral type I 5'-deiodinase. The fourth group received the same treatment as the hypothyroid birds but also received daily subcutaneous injections of 3,5,3'-triiodo-L-thyronine (T_3_; Sigma; 10 μg/100 g body weight dissolved in 0.9% NaCl, 20 mM NaOH) and is referred to as the T_3_-treated group (n = 7). TN, CA and hypo ducklings also received daily subcutaneous injections of the vehicle solutions.

At the end of the treatment, ducklings were euthanized and blood was collected on heparin. Plasma was separated and frozen for subsequent determinations of plasma levels of triglycerides (GPO-Trinder kit from Sigma), non-esterified fatty acids (Wako-Unipath kit) and total T_3 _(coat-a-count RIA kit from ICN Biomedicals). Tissues including liver and red gastrocnemius muscle were rapidly sampled and frozen in liquid nitrogen.

The experimental protocols were approved by the French Ministry of Agriculture Ethics Committee and were in accordance with the guiding principles of the French Department of Animal and Environmental Protection for the care and use of laboratory animals.

### Relative abundance of avUCP transcripts

Avian uncoupling protein (avUCP) expression was assessed by reverse transcription-polymerase chain reaction (RT-PCR). Total RNA was isolated from 0.1 g portions of frozen tissue, as described by Rey et al. [27], using a Trizol solution (Euromedex, France). RNA concentrations were estimated by measuring the absorbance at 260 nm, and RNA integrity was checked by gel electrophoresis.

Reverse transcription (RT) of 1 μg total RNA was performed as described previously [27] using 200 UI M-MLV-RT (Promega, France), 2 μg/mL Poly T primers (Invitrogen, France), 1 mM deoxyribonucleotides and 25 UI RNAsin (Promega, France). PCR was then performed with one-tenth of the RT reaction in a total volume of 50 μL containing 0.2 mM dNTPs (Eurobio), 1 μM forward (5'-GTGGATGCCTACAGGACCAT-3') and 1 μM reverse (5'-ATGAACATCACCACGTTCCA-3') primers of avUCP, 2.5 U of Taq DNA polymerase (Eurobio), Taq reaction buffer (Eurobio) and 1.5 mM MgCl_2 _in a Hybaid thermocycler (Ashford). The reaction started with an initial denaturation at 94°C for 2 min followed by 27 cycles of denaturation at 94°C for 45 s, annealing at 65°C for 60 s and extension at 72°C for 60 s. A final extension was performed at 72°C for 10 min. RT-PCR of GAPDH mRNA was performed following the same protocol using forward 5'-TTTGGCCGTATTGGCCGCCGCCT-3' and reverse 5'-CAGCAGCCTTCACTACCCTC-3' primers (annealing 58°C, 24 cycles, 766 bp PCR product). The amplified products were easily separated according to their size on 1.5% agarose gels (Sigma) stained with ethidium bromide (0.5 mg/mL, Sigma). Relative intensities of bands were assessed using a Kodak Digital Science 1D Image Analysis Software. The target cDNA-to-GAPDH ratio was then used as a relative estimate of mRNA abundance. PCR were performed in duplicate and values were averaged. A longer (780 bp) PCR product of duck UCP was obtained for sequencing using forward 5'-GCGGTCGACATCACCTTCCCGCTGGACAC-3' and reverse 5'-CATGTCGACGTTCCAGGATCCGAGTCGC-3' primers. Products were purified with a Cleanix Talent kit according to the manufacturer's instructions and sequenced on both strands (Genoscreen, Lille, France).

### Isolation of mitochondria

Intermyofibrillar mitochondria were isolated from gastrocnemius muscle by homogenization, protease digestion and differential centrifugation in ice-cold isolation buffer (100 mM sucrose, 50 mM Tris base, 50 mM KCl and 5 mM EDTA; adjusted to pH 7.4 with HCl at 4°C) as previously described for birds [27] and suspended in a minimal volume of final medium containing 250 mM sucrose, 1 mM EGTA and 20 mM Tris base, pH 7.4. Liver mitochondria were isolated by differential centrifugation in ice-cold isolation buffer (250 mM sucrose, 20 mM Tris HCl and 1 mM EGTA, pH 7.4). Protein concentrations of mitochondrial suspensions were determined using the Biuret method with bovine serum albumin (BSA) as the standard. Mitochondrial content (mg mitochondrial protein per g muscle) was calculated according to Rey *et al. *[27] from the activity of cytochrome c oxidase (Barré *et al. *[37]), which was determined polarographically in isolated mitochondria and in skeletal muscle homogenates.

### Mitochondrial oxygen consumption rates and mitochondrial membrane potential

Oxygen consumption and mitochondrial membrane potential were measured simultaneously, as previously described for birds by Talbot et al. [25]. The oxygen consumption of mitochondria in the presence of oligomycin (to inhibit the F1-F0 ATP synthase) is proportional to the rate at which protons leak across the mitochondrial inner membrane. The kinetic response of the proton conductance to its driving force (proton-motive force) can therefore be measured as the relationship between oxygen consumption and mitochondrial membrane potential (assuming no slip reaction in the mitochondrial proton pumps) when the potential is varied with increasing doses of electron transport chain inhibitors [21,25,38,39]. Oxygen consumption was measured using a 3.5-mL, Clark-type oxygen electrode (Rank Brothers Ltd, France) maintained at 38°C and calibrated with air-saturated assay medium (120 mM KCl, 5 mM KH_2_PO_4_, 2 mM MgCl_2_, 3 mM Hepes, 1 mM EGTA and 0.3% (w/v) BSA, pH 7.4), which was assumed to contain 402 nmol O/mL. Electrode linearity was checked routinely by following the uncoupled respiration rate in the presence of 0.4 μM carbonyl cyanide p-trifluoromethoxyphenylhydrazone (FCCP) from 100-0% air saturation. Membrane potential was determined simultaneously using the potential-dependent probe triphenylmethyl phosphonium cation (TPMP^+^) (7).

Mitochondria (0.35 mg protein/mL for muscle and 1 mg/mL for liver) were incubated at 38°C in assay medium containing 5 μM rotenone (Sigma R8875), 1 μg/mL oligomycin (Sigma O4876) and 65 ng/mL nigericin (Sigma N7143) to collapse the difference in pH across the mitochondrial inner membrane. Rotenone, oligomycin and nigericin solutions were prepared in ethanol. The TPMP^+ ^electrode was calibrated with four sequential additions of TPMP^+ ^(Sigma 130079), up to 2 μM, and 5 mM succinate (Sigma S3674) was then added to start the reaction. Respiration and membrane potential were progressively inhibited through successive steady states by addition of malonate up to 3 mM. At the end of each run, 2 μM FCCP (Sigma C2920) was added to dissipate the membrane potential and release all TPMP^+ ^back into the medium; post-dissipation measurements allowed for the correction for any small electrode drift. Extra- and intra-mitochondrial TPMP^+ ^concentrations were determined and the membrane potentials were calculated using the Nernst equation; the mitochondrial matrix volume was taken into account, and binding correction factors for TPMP^+ ^were assumed to be 0.42 and 0.35 μL/mg of protein for liver and muscle, respectively [21,40].

Where indicated, exogenous superoxide was generated using xanthine (50 μM, Sigma X0626) and xanthine oxidase (8 mU/3.5 mL, Sigma X4376). A stock solution of 0.35 mM xanthine was stored at -20°C whereas xanthine oxidase (1 U/mL) was prepared daily. Xanthine and xanthine oxidase were prepared in assay medium buffered at pH 7.4. GDP (Sigma G7127) was prepared daily in assay medium at a concentration of 125 mM. Palmitate (Sigma P9767) was prepared in absolute ethanol at a concentration of 25 mM. Whether added alone or in combination, xanthine/xanthine oxidase, GDP and/or palmitate were always added in the assay medium before TPMP^+ ^calibration.

### Measurement of mitochondrial H_2_O_2 _production

The rate of mitochondrial H_2_O_2 _release was assessed following the linear increase in fluorescence (λ_ex _312 nm and λ_em _420 nm) due to the oxidation of homovanilic acid (HVA) by H_2_O_2 _in the presence of horseradish peroxidase (HRP) using a SFM-25 fluorometer (Kontron), as described previously [41]. Reaction conditions were as follows: 0.05 mg of muscle mitochondrial protein per mL and 0.15 mg of liver mitochondrial per mL, 6 U/mL HRP, 0.1 mM HVA and 5 mM succinate; reactions were performed in the same incubation buffer used for oxygen consumption measurements. For technical reasons, mitochondrial H_2_O_2 _production was measured at 30°C. When succinate is used as substrate of the respiratory chain, superoxide production essentially occurs on the matrix side of the inner membrane during the reverse electron transport from complex II to complex I [42]. Generated superoxides are converted either spontaneously or by the mitochondrial mangano-superoxide dismutase (MnSOD) into H_2_O_2 _that can leak across membranes and be measured in the medium assay. The fluorescence units were then converted into an H_2_O_2 _concentration after addition of known concentrations of H_2_O_2 _to establish the standard concentration curve. In order to calculate the fraction of electrons which reduce O_2 _to H_2_O_2 _at the mitochondrial level, mitochondrial oxygen consumption was then measured under the same conditions (buffer, substrate concentration, temperature) and calculated according to [43].

### Measurement of antioxidant enzyme activity (GPx and SOD)

Enzyme activity was determined as previously described for birds [27]. Briefly, portions of frozen gastrocnemius muscle were homogenized with a potter Elvehjem at 4°C, in buffer containing 100 mM KH_2_PO_4_, 1 mM DTT and 2 mM EDTA at pH 7.4. After centrifugation (3000 g for 5 min), the supernatant was used for enzymatic assays. Superoxide dismutase (SOD) activity was assayed by monitoring the rate of acetylated cytochrome *c *reduction by superoxide radicals generated by the xanthine-xanthine oxidase system [44]. One activity unit of SOD was defined as the amount of enzyme that inhibits the rate of acetylated cytochrome *c *reduction by 50%. To distinguish mangano-SOD (MnSOD), which is exclusively located in the mitochondrial matrix, from cuprozinc-SOD (Cu,Zn-SOD), which is primarily located in the cytosol, SOD activity was determined after incubation with 1 mM NaCN. At this concentration, cyanide inhibits the Cu,Zn isoform of the enzyme but does not affect the Mn isoform [44]. The assay for total activity of glutathione peroxidase (GPx) coupled the reduction of cumene hydroperoxide to the oxidation of NADPH by glutathione reductase, and this coupled reaction was monitored at 340 nm [45]. All enzyme activities are expressed as U/g muscle.

### Statistical analysis

Data are expressed as means ± SEM. Statistical significance was determined by one or two-way analysis of variance (ANOVA) for independent (when comparisons were made between experimental groups) or repeated (when comparisons were made between respiratory states of mitochondria within the same experimental groups) values followed by PLSD tests or paired Student's *t *tests, respectively. Differences were considered significant at P < 0.05.

## Results

### Growth curves, circulating total T_3 _and lipids, tissue mass and mitochondrial content

**Figure 1:**
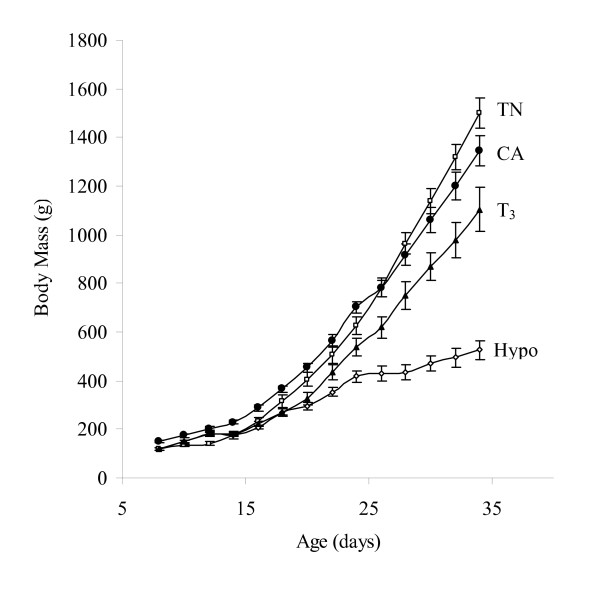
**Growth curves of thermoneutral controls (TN), cold-acclimated (CA), hypothyroid (hypo) or T_3_-treated ducklings**. Values are mean ± S.E.M. from 7-9 birds per group.

As shown in Figure 1, rearing ducklings in the cold slightly reduced growth despite increased food intake (+30%, P < 0.05). Birds given PTU showed rapidly hindered growth, and by the time of killing body mass was only one-third of that of TN controls (Table 1). T_3 _treatment to ducklings given PTU rapidly restored but did not normalize growth. Cold acclimation (CA) led to increased circulating levels of total T_3 _(+120%, P < 0.05), while ducklings receiving PTU were clearly hypothyroid (-90% of circulating total T_3 _vs. controls, P < 0.05) and those receiving PTU+T_3 _were hyperthyroid (Table 1). Hypothyroid ducklings also exhibited marked circulating hyperlipidemia which was reversed by T_3 _treatment.

**Table 1:** Body and relative organ masses, food intake, circulating levels of total T_3_, triglyceride, NEFA and tissue mitochondrial content in thermoneutral controls, cold-acclimated, hypothyroid or T_3_-treated ducklings.

	Hypo	TN	CA	T_3_
Body mass, kg	0.50 ± 0.03^***ac***^	1.63 ± 0.08	1.51 ± 0.09	1.36 ± 0.07^***ab***^
				
Food Intake, kcal/kg^0.75^/day	88 ± 5^***ac***^	106 ± 3	139 ± 4^***ab***^	146 ± 8^***ab***^
Tissue mass, g/100 g				
Gastrocnemius muscle	1.5 ± 0.1	1.4 ± 0.03	1.5 ± 0.1	1.2 ± 0.1
Liver	8.3 ± 0.4^***ac***^	3.2 ± 0.2	3.3 ± 0.1	4.9 ± 0.3^***abc***^
Plasma level:				
T_3_, nM	0.17 ± 0.02^***ac***^	1.7 ± 0.2	3.8 ± 0.6^***a***^	36.1 ± 1.8^***abc***^
Triglyceride, mM	8.4 ± 0.2	2.7 ± 0.3^***b***^	1.7 ± 0.2^***b***^	1.5 ± 0.3^***b***^
NEFA, mM	1.99 ± 0.15^***ac***^	0.71 ± 0.03	0.93 ± 0.05^***a***^	0.81 ± 0.15^***b***^
				
Mitochondrial content, mg/g				
Gastrocnemius muscle	16.0 ± 1.1^***ac***^	32.6 ± 1.8	45.1 ± 2.3^***a***^	49.8 ± 4.2^***ab***^
Liver	117 ± 17^***ac***^	225 ± 16	243 ± 13	173 ± 11^***abc***^

The relative liver mass (g/100 g) was significantly increased in hypothyroid ducklings (+159% vs. controls, P < 0.05), an effect that was partially reversed in ducklings receiving T_3 _injection (+53% vs. controls, P < 0.05). The liver mass was not affected by cold acclimation (Table 1). Gastrocnemius muscle relative to body weight remained unaffected in all three experimental groups (Table 1).

Skeletal muscle mitochondrial content was increased after cold acclimation or T_3 _treatment compared with control ducklings but was reduced by hypothyroid status (Table 1). Liver mitochondria content, by contrast, was only affected by pharmacological changes in thyroid status.

### Expression of avUCP mRNA

With different sets of primers, expression of the avUCP transcript was detected by RT-PCR in red gastrocnemius muscle, but no expression was found in the liver in any experimental group (data not shown). The nucleotide sequence of duck UCP (780 bp) showed a strong structural homology with the other avian UCPs described previously (91% with chicken and turkey, 93% and 86% with king penguin and hummingbird, respectively) [21,24,46]. Sequence homology was lower with rat UCP1 (62%), UCP 2 (72%) and UCP3 (78%).

**Figure 2:**
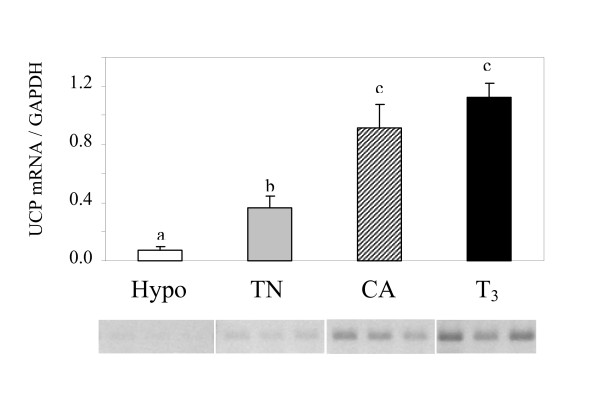
**Relative abundance of UCP mRNA in red gastrocnemius muscle from thermoneutral controls (TN), cold-acclimated (CA), hypothyroid (hypo) or T_3_-treated (T_3_) ducklings**. RT-PCR results of three birds per group are illustrated. Bar values are means ± S.E.M. from six birds per group. Bars with different letters are significantly different (P < 0.05).

Figure 2 shows that in red gastrocnemius muscle, the relative abundance of UCP mRNA was reduced by hypothyroidism (-81%, P < 0.05) as compared with thermoneutral controls but was markedly increased in CA (+150%, P < 0.05) and T_3_-treated ducklings (+208%, P < 0.05).

### Basal proton conductance of mitochondria

**Figure 3:**
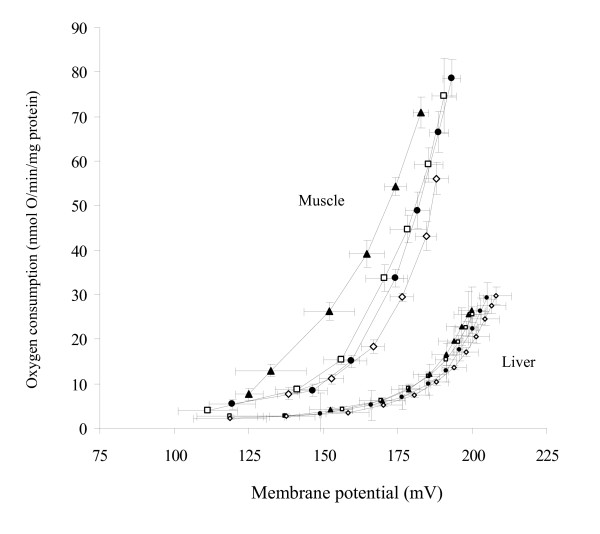
**Basal proton leak in red gastrocnemius muscle and liver mitochondria from thermoneutral controls (open square), cold-acclimated (black cycle), hypothyroid (open diamond) or T_3_-treated ducklings (black triangle)**. Respiration and membrane potential were measured at 38°C under basal conditions (absence of UCP activators) in the presence of 0.3% FAF-BSA, 2 μM TPMP^+^, 65 ng/mL nigericin, 1 μg/mL oligomycin, 5 μM rotenone and 5 mM succinate. Pulses of malonate were added up to 3 mM to induce variations in membrane potential. Values are means ± S.EM. from 5-8 independent determinations per group.

Figure 3 shows the kinetic response of the basal proton leak rate to its driving force, membrane potential, in mitochondria isolated from duckling gastrocnemius muscle and liver under control conditions. The proton conductance of skeletal muscle mitochondria was not different between TN controls and CA ducklings, while it tended to be reduced in hypothyroid birds and was higher in T_3_-treated birds. Hence, the basal proton conductance of muscle mitochondria calculated at 170 mV (assuming a constant stoichiometry of 6 H^+^/O for succinate) was significantly increased by 38% (P < 0.01) and 67% (P < 0.001) in T_3_-treated ducklings (1.75 ± 0.14 nmol H^+^.min^-1^.mV^-1^.mg protein^-1^) as compared with control (1.27 ± 0.11 nmol H^+^.min^-1^.mV^-1^.mg protein^-1^) and hypothyroid (1.05 ± 0.01 nmol H^+^.min^-1^.mV^-1^.mg protein^-1^) birds, respectively. No major change in proton conductance was observed in any liver mitochondrial preparation.

### Assessment of a functional UCP in isolated mitochondria

**Figure 4:**
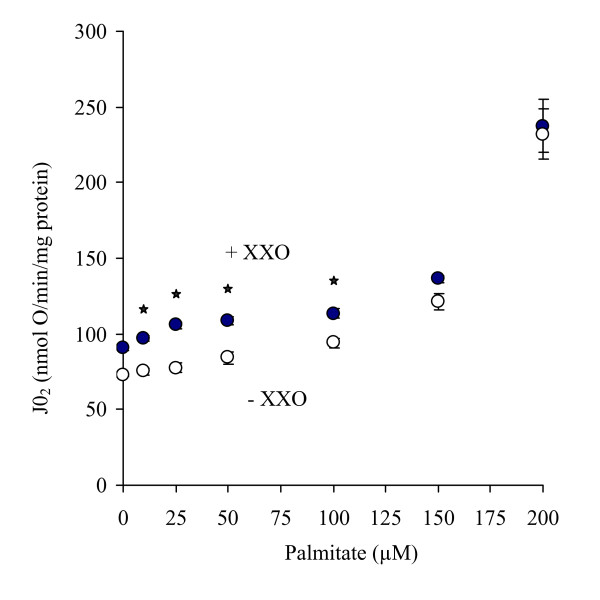
**Effect of increasing doses of palmitate on non-phosphorylating respiration of skeletal muscle mitochondria from T_3_-treated ducklings in the presence (dark circles) or absence (open circles) of xanthine/xanthine oxidase (XXO)**. Mitochondria were isolated from T_3_-treated ducklings and respiration was initiated with 5 mM succinate in the presence of 0.3% FAF-BSA. Values are means ± SEM from 5-6 determinations. * indicates a significant (P < 0.05) interaction between the dose of palmitate and the addition of XXO implying a higher stimulation of XXO at a given palmitate concentration.

We used the common property of a superoxide activated proton conductance shared by mammalian UCPs [18] and avUCP [21,27,47] to detect a functional UCP in duckling mitochondria *in vitro*. Because the superoxide activation of these UCPs only occurs in the presence of free fatty acid (FFAs) and is potently inhibited by nucleoside di- and triphosphates, it was important to determine the optimal conditions that would allow such a determination. As shown in Figure 4, increasing doses of palmitate stimulated mitochondrial non-phosphorylating respiration. In the presence of xanthine and xanthine oxidase (XXO), the rate of non-phosphorylating respiration was further stimulated at low doses of palmitate (P < 0.05), implying a higher stimulation of XXO at a given palmitate concentration. This effect of XXO at low doses of palmitate was reversed by GDP, unraveling a UCP activity. In contrast, large doses of palmitate masked the XXO stimulation. Further, large doses of palmitate stimulated a non-phosphorylating mitochondrial respiration that was not sensitive to GDP. Hence, irrespective of the presence of XXO, high concentrations of fatty acids target non-UCP dependent leak mechanisms, which would involve other mitochondrial transporters such as adenine nucleotide translocase (Roussel et al., 1998). For these reasons, we selected low doses of palmitate (25 μM) for further analyses.

**Figure 5:**
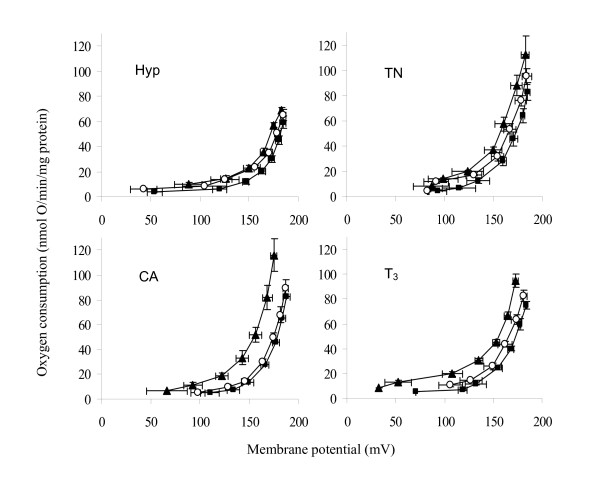
**Effect of superoxide plus palmitate in the absence (black triangle) or presence (open cycle) of GDP on basal proton conductance (black square) of red gastrocnemius muscle mitochondria isolated from thermoneutral controls (TN), cold-acclimated (CA), hypothyroid (hypo) or T_3_-treated ducklings**. Proton conductance was assessed by simultaneous measurement of membrane potential and oxygen consumption. Superoxide generation was obtained by addition of 50 μM xanthine (X) and 8 mU/3.5 mL xanthine oxidase (XO). When observed, increase in proton conductance was reversed by 1 mM GDP. All measurements were conducted with 5 mM succinate as substrate, 5 μM rotenone, 1 μg/mL oligomycin, 65 ng/mL nigericin, 0.3% FAF-BSA and 25 μM palmitate. Mitochondria from CA or T_3_-treated ducklings showed a much larger GDP sensitive XXO-activated proton conductance compared with TN or hypothyroid birds in which XXO had no major effect. Data are means ± S.E.M. from 5 to 11 independent determinations.

Figure 5 shows the kinetic response of the proton leak rate to its driving force in skeletal muscle mitochondria from ducklings in the presence of 0.3% BSA (to clamp the free fatty acid concentration) and 25 μM palmitate. Addition of XXO increased the respiration rate of mitochondria from both hyperthyroid and CA ducklings, while it lowered membrane potential. This increase in the proton conductance of the mitochondrial inner membrane was inhibited by GDP, a known UCP inhibitor.

The activation of proton conductance by the addition of XXO and 25 μM palmitate was less pronounced in muscle mitochondria from control or hypothyroid ducklings than those from cold-acclimated or hyperthyroid birds (Figure 5). The difference between XXO (filled triangles) and XXO plus GDP (open circles) curves represents the oxygen that the mitochondria consumed to counteract the activity of the proton leak catalyzed by UCP. In this condition, the GDP-dependent oxygen consumption of muscle mitochondria calculated at 170 mV was significantly higher in hyperthyroid ducklings (35 ± 8 nmol O.min^-1^.mg protein^-1^, P < 0.05) than in control (14 ± 7 nmol O.min^-1^.mg protein^-1^) or hypothyroid birds (8 ± 3 nmol O.min^-1^.mg protein^-1^). Given the value calculated in CA ducklings (30 ± 8 nmol O.min^-1^.mg protein^-1^), it appears that the activity of avUCP in mitochondria is related to the relative abundance of the avUCP transcript in skeletal muscle (Figure 2).

### Mitochondrial H_2_O_2 _production

**Figure 6:**
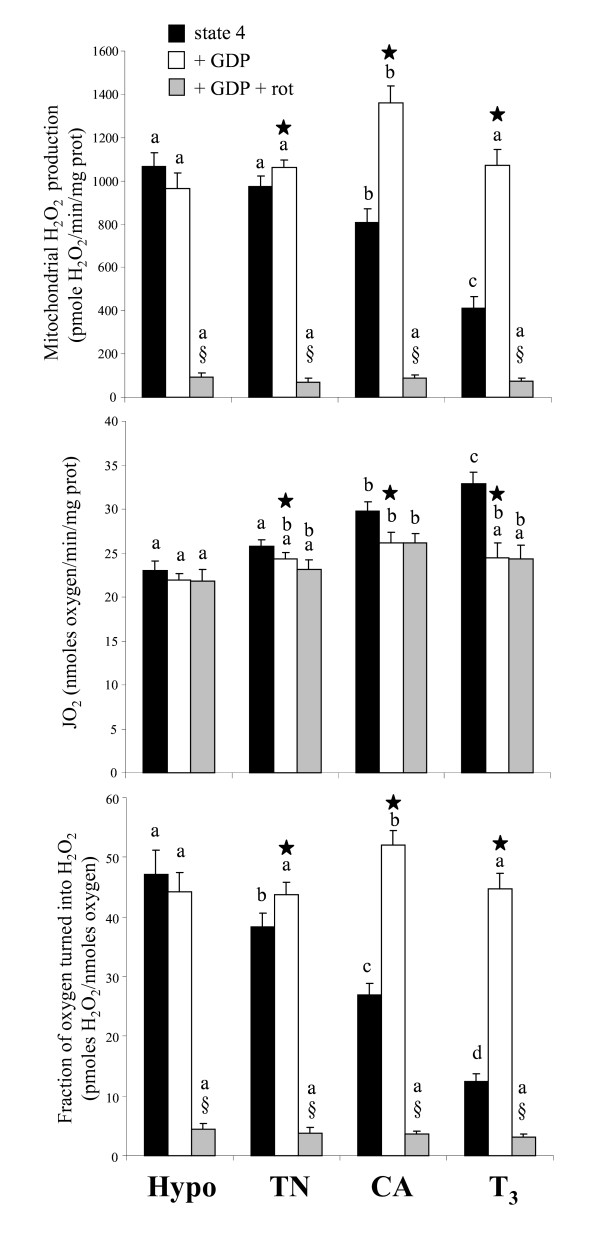
**Release of H_2_O_2 _by intermyofibrillar mitochondria isolated from red gastrocnemius muscle of thermoneutral controls (TN), cold-acclimated (CA), hypothyroid (hypo) or T_3_-treated (T_3_) ducklings**. Oxygen consumption (JO_2_) was measured in parallel, and H_2_O_2 _production was expressed per oxygen consumed. Measurements were performed in the presence of 0.3% FAF-BSA and 5 mM succinate without (black columns) or with 1.5 mM GDP (open columns) or with 1.5 mM GDP + 5 μM rotenone (grey columns). Within an experimental condition of respiration (succinate, succinate + GDP or succinate + GDP + rotenone), bars with different letters are significantly different (P < 0.05). * indicates a significant effect of GDP (P < 0.05); § indicates a significant effect of rotenone (P < 0.05).

In order to investigate potential functional consequences of the presence of avUCP in duckling skeletal muscle mitochondria, we measured the rate of endogenous H_2_O_2 _generation *in vitro *in respiratory state 4 with succinate as the substrate. Figure 6 shows that the endogenous ROS production was similar in mitochondria from hypothyroid or control ducklings. ROS production was lower in mitochondria from CA ducklings (-17% vs. controls, P < 0.05) and was much lower in mitochondria from T_3_-treated ducklings (-58% vs. controls, P < 0.001).

Most interestingly, addition of GDP at a concentration likely to saturate all GDP-binding sites (1.5 mM), markedly increased H_2_O_2 _production in mitochondria from CA (+68% above state 4, P < 0.05) or T_3_-treated (+159% above state 4, P < 0.05) ducklings. A minor though significant effect of GDP (+8%, P < 0.05) was noticed in controls, but no effect of GDP was observed with mitochondria from hypothyroid ducklings. It is also worth noting that GDP-stimulated H_2_O_2 _production was brought back at or above the level measured in controls. Addition of rotenone strongly inhibited mitochondrial H_2_O_2 _production in all groups, indicating that most of the H_2_O_2 _production occurred at the level of complex I. Because H_2_O_2 _production and O_2 _consumption were measured in the same conditions (buffer, substrate concentration, temperature), the fraction of O_2 _turned into H_2_O_2 _instead of being reduced to water was calculated. As shown in Figure 6, endogenous H_2_O_2 _release per unit of oxygen consumed was the highest in mitochondria from hypothyroid ducklings and the lowest in those from T_3_-treated birds, and there was a gradual decrease in H_2_O_2 _release between groups: hypo>controls>CA>T_3_-treated. Again, the addition of GDP markedly increased the mitochondrial H_2_O_2 _production of CA or hyperthyroid birds up to or above the level of control or hypothyroid ducklings, while rotenone inhibited most of the H_2_O_2 _release.

H_2_O_2 _release from liver mitochondria was higher than that of muscle mitochondria in all groups (81 ± 6 vs. 38 ± 2; 79 ± 3 vs. 47 ± 4; 57 ± 2 vs. 12 ± 1 and 89 ± 20 vs. 26 ± 3 pmol H_2_O_2 _per nmol oxygen, in controls, hypothyroid, T_3_-treated and cold acclimated birds, respectively, ANOVA P < 0.05). There was no significant effect of cold exposure or hypothyroidism, while H_2_O_2 _release was slightly decreased by hyperthyroidism (-23%, P < 0.05). Contrary to muscle mitochondria, GDP did not activate hepatic ROS production even in T_3_-treated ducklings (data not shown).

**Figure 7:**
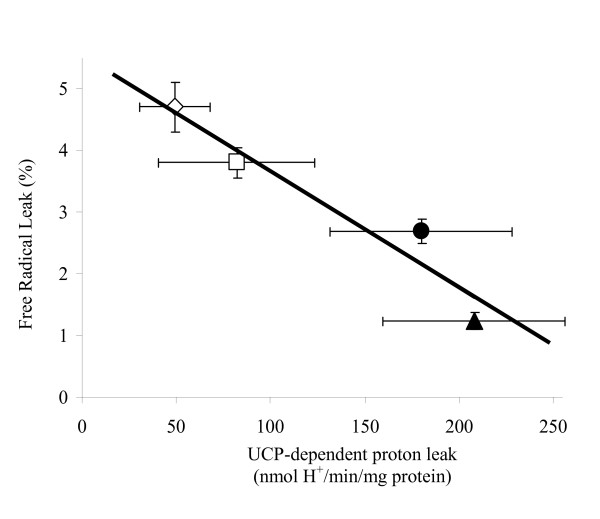
**Relationship between the fraction of O_2 _turned into H_2_O_2 _instead of being reduced to water (free radical leak) and the UCP-dependent mitochondrial proton leak in mitochondria extracted from thermoneutral controls (open square), cold-acclimated (black cycle), hypothyroid (open diamond) or T_3_-treated ducklings (black triangle)**. UCP-dependent proton leak was calculated from the difference between XXO-activated and GDP-sensitive respiration at 170 mV, assuming a stoichiometry of 6. There was a linear relationship between both parameters (y = -0.019x + 5.567, R^2 ^= 0.92). Values are means ± S.E.M.

The fraction of O_2 _turned into H_2_O_2 _instead of being reduced to water (basal electron leak) was also plotted against the UCP-dependent mitochondrial proton leak as calculated above from the difference between XXO-activated and GDP-sensitive respiration at 170 mV. As shown in Figure 7, there was a linear reduction (R^2 ^= 0.92, P < 0.05) in basal electron leakage when UCP-dependent proton leak increases, further emphasizing a straight correlation between a functional of avUCP and ROS production.

### Antioxidant enzyme activity

In our experiments, ROS measurement reflected the balance between mitochondrial H_2_O_2 _production and elimination by antioxidant systems. There was no significant difference in cytosolic (Cu,Zn) SOD, mitochondrial mangano-superoxide dismutase (MnSOD) or GPX activities between thermoneutral controls and CA ducklings (Table 2). Hypothyroidism led to a reduction in SOD activities while T_3_-treatment did not completely restore control levels. GPX activity was not influenced by thyroid status. These observations exclude the possibility that the drop in H_2_O_2 _production measured in mitochondria from CA or T_3_-treated birds resulted from a more effective antioxidant system.

**Table 2:** Activities of cytochrome-c oxidase and antioxidant enzymes in skeletal muscle.

Enzymes	Hypo	TN	CA	T_3_
COX	8 ± 2 *	31 ± 4	35 ± 5	23 ± 3
SOD				
total	1164 ± 112 *	2134 ± 222	2039 ± 250	1506 ± 114 *
Mn	729 ± 59 *	1048 ± 115	1011 ± 88	956 ± 121
Cu,Zn	435 ± 151 *	1086 ± 204	1028 ± 279	550 ± 121 *
GPX	1.17 ± 0.13	1.39 ± 0.22	1.56 ± 0.16	1.60 ± 0.16

Enzyme activities were measured in red gastrocnemius muscle from thermoneutral control, cold-acclimated, hypothyroid or T_3_-treated ducklings. Cytochrome-c oxidase (COX) was measured polarographically in tissue homogenates. Superoxide dismutase and glutathione peroxidase were measured spectrophotometrically in tissue homogenates.

## Discussion

The protocol of cold acclimation used in the present study was designed to induce muscle adaptive thermogenic processes described in previous studies [35,36]. The cold-induced reduction in growth despite increased food intake is in agreement with previous studies [48] and indicates a high energy expenditure for regulatory thermogenesis. This is reinforced by a cold-induced increase in muscle mitochondria content (+38%, Table 1) that may help improve cellular ATP generation. In parallel, while birds exposed to cold demonstrated higher hepatic lipogenic capacities [49], our data show a reduction in circulating triglycerides compared to thermo-neutral controls and also show a higher level of plasmatic non-esterified fatty acid (NEFA, Table 1), suggesting increased tissue uptake of plasma triglycerides through lipoprotein lipase and activated lipolysis. The increased availability of fatty acids in the cold, also observed by others [23-26], supports the hypothesis that fatty acids play a major role in acclimation to cold both as substrates of muscle activity [49] and putatively as uncouplers of mitochondrial oxidative phosphorylation [36,50]. Further, fatty acids may also contribute to the activation of avUCP expression by analogy with mammals in which the expression of muscle UCPs mainly depends on NEFA activation of PPARs (peroxisome proliferator-activated receptors) through their interaction with the UCP promoter [51]. Because CA ducklings also present slight hyperthyroidism compared to controls (Table 1), in agreement with data in mammals and birds [23,52,53], we cannot rule out the possibility that the increased expression of avUCP in the cold also involves changes in thyroid status [54,55].

Treatment with thyroid inhibitors or T_3 _clearly modified the thyroid status of ducklings kept at thermoneutrality, as reflected by changes in circulating T_3_, and also affected body mass (Table 1 and Figure 1) and avian UCP expression (Figure 2), which is in agreement with previous studies in birds [55] and in mammals [56]. Interestingly, ducklings given PTU were characterized by a very weak abundance of avUCP mRNA despite a dramatic circulating hyperlipidemia (+210% triglyceride and +180% NEFA vs. controls, Table 1). By contrast, T_3_-treated ducklings were characterized by a marked increase in avUCP mRNA (Figure 2) levels with no significant changes in circulating triglycerides and NEFA (+14%, Table 1) as compared with control birds. These results suggest that, in our models, avUCP expression is mainly controlled by thyroid status rather than by plasma levels of triglycerides or fatty acids.

Next, we explored the functionality of avUCP measuring the inner membrane proton leakage as determined by the relationship between respiration rate and membrane potential. We show that in the absence of specific activators, the abundance of avUCP mRNA is not related to changes in mitochondrial basal proton leak except for in extreme conditions such as hyperthyroidism (Figure 3). It follows that in basal *in vitro *conditions, avian UCP is not active and does not affect mitochondrial proton leak significantly. Similarly, overexpression of avUCP in yeast did not increase mitochondrial membrane permeability in the absence of activators [30]. In hyperthyroid ducklings, the higher basal proton leak may indicate that either a given protein level is required to observe a leak effect or that other mechanisms such as T_3_-induced changes in membrane phospholipid composition also contribute to membrane leakiness.

The present results show that a GDP-sensitive stimulation of mitochondrial proton leakage was obtained in the presence of known avian and mammals UCP *in vitro *co-activators, e.g., exogenous superoxide generated by the enzymatic reaction of xanthine and xanthine oxidase (XXO) and an adequate amount of NEFA [18,21]. Indeed, in the present study, activation of a mitochondrial proton leak by exogenous superoxide was only measurable when an optimal amount of palmitate was added (Figure 4). At higher concentrations of fatty acids, increased leakiness through the inner mitochondrial membrane becomes insensitive to GDP, representing an uncoupling effect that can be catalyzed by other mitochondrial transporters such as ANT [50]. We, thus, suggest that the amount of fatty acids is a critical parameter to consider and may explain discrepancies between the present data and a previous study that did not find a XXO-induced and GDP-sensitive activation of proton leakage in avUCP-transfected yeasts [30].

In the given experimental conditions, the avUCP-dependent proton leak clearly varied in relation to the relative abundance of avUCP mRNA in skeletal muscle, i.e., increased in muscle mitochondria from CA and T_3_-treated ducklings compared to TN controls, and was absent in muscle mitochondria from hypothyroid ducklings (Figure 5) and in all liver mitochondrial preparations. Similar results have also been reported in penguins following adaptation to cold water [25]. Stimulation of proton leakage by exogenous superoxide in the presence of FFA was only observed in cold acclimated penguins with elevated expression of muscle UCP. By contrast, it was shown that activation of proton conductance through UCP3 in mouse skeletal muscle mitochondria is absent in mitochondria from UCP3 knockout mice. This observation strongly suggests that the superoxide-activated, GDP-sensitive proton conductance in duckling muscle mitochondria was mediated by avUCP. This result is further underlined by the fact that superoxide plus palmitate did not affect mitochondrial proton leakage in mitochondria from the liver, a tissue with no detectable expression of avUCP transcripts. It, therefore, appears that a given level of expression of avUCP and adequate stimulations are required to induce a detectable change in proton leak activity related to UCP activity at the mitochondrial level.

Thyroid hormone administration and cold-induced hyperthyroidism are also known to increase metabolic rate and induce oxidative stress [57]. There are two main mechanisms by which organisms may prevent oxidative stress: (i) by increasing antioxidant defences in tissues, and/or (ii) by reducing mitochondrial ROS production. As far as our study is concerned, the lack of a significant alteration of antioxidant enzyme activity (Table 2) suggests that a controlled reduction of mitochondrial ROS generation through the activation of avUCP would allow CA or T_3_-treated ducklings to prevent their skeletal muscle from oxidative damage. Activation of avUCP would generate a mild uncoupling that may decrease proton motive force (Figure 5), reducing local oxygen tension and attenuating superoxide production [3,8,10,19]. It was recently hypothesized that this phenomenon also occurs in birds since the fasting-induced rise in UCP expression was associated with a lower production of ROS [33]. Present results confirm and extend this notion as we show that an up-regulation of avUCP in skeletal muscle induced by either cold exposure or hyperthyroidism was clearly associated with a GDP-dependent limitation of H_2_O_2 _production (Figure 6, 7). Conversely, H_2_O_2 _production was high and insensitive to GDP with a hypothyroidism-induced drop in avUCP. Further, no rise in H_2_O_2 _production was observed with the addition of GDP in liver mitochondria in which no activation by XXO plus palmitate was detected.

## Conclusions

Taken together, our data show that avian UCP plays a role as a modulator of ROS production in skeletal muscle mitochondria. This antiradical function of avUCP would be of particular importance in preventing cellular damage under hyper metabolism linked to high cellular oxidative activity and a high degree of reduction of the respiratory chain as occurs in hyperthyroidism and chronic exposure to cold.

## Abbreviations

ANT: Adenine Nucleotide Transporter; GDP: Guanosine Di phosphate; GPX: Glutathione Peroxidase; FFA: Free Fatty Acid; NEFA: Non-Esterified Fatty Acids; PPAR: Peroxysome Proliferator-Activated Receptors; PTU: 6-n-propyl-2-thiouracil; ROS: Reactive Oxygen Species; SOD: Superoxide Dismutase; UCP: Uncoupling Protein; XXO: Xanthine and Xanthine Oxidase.

## Authors' contributions

BR carried out the experimental design. DR, JLR and BR were in charge of the muscle mitochondrial extraction, mitochondrial respiration, proton leak measurements and data analysis. CD conceived the study and participated in its design and coordination. CD, BR, MB and SS were in charge of ROS determination and molecular approaches. CR and BS contributed to mitochondrial respiration, enzyme activity and plasma assays. DD measured antioxidant enzyme activities. All authors read and approved the final manuscript.
